# The Works of John Hunter, F.R.S.

**Published:** 1839-07

**Authors:** 


### ART. VIII.

1. *The Works of* JOHN HUNTER, F.R.S.; *with Notes by* JAMES F. PALMER. In four Volumes. Vol. III. *A Treatise on the Blood, Inflammation, and Gunshot Wounds.—London*, 1837. 8vo, pp. 586.

2. *A Treatise on Inflammation.* By JAMES MACARTNEY, M.D. F.R.S. F.L.S. M.R.I.A. &c. &c.—*London*, 1838. 4to, pp. 214.

3. *Illustrations of the Elementary Forms of Disease.* By ROBERT CARSWELL, M.D., Professor of Pathological Anatomy in University College, London, &c. Fasciculus, XII. *Inflammation.—London*, 1838. Folio, pp. 8.

4. *Teoria della Flogosi, di* GIOVANNI RASORI.—*Livorno*, 1837. 8vo, pp. 260.

*Theory of Inflammation.* By J. RASORI.—*Leghorn*, 1837.

IF any pathologist were asked what he regarded as the most complete department of his science, that most reduced to general principles, and consequently furnishing the most certain rules for the therapeutic art, he would probably name, without much hesitation, the subject of inflammation. And yet a very little attention must convince the discerning practitioner how imperfect is even this best-understood department of pathology; and how liable are its precepts to fail of success, even when the apparent simplicity of the case would lead him to place the most implicit reliance upon them. Take, for example, the local treatment of an acute sthenic inflammation; how purely empirical is it! One surgeon will apply cold, another warmth; one will use astringent or strong stimulants, whilst another finds more satisfaction in emollients and sedatives. And, after all, it will probably be by the feelings of the patient, and the remedial efficacy of the means, that the continuance of any particular plan will be decided on. If we turn to the pages of authors on the theory of inflammation, we shall no longer feel surprised at the uncertainty of the therapeutic rules with which they supply us. Few subjects have been more keenly debated; few debates have ended in a less satisfactory conclusion. To what cause can this result be attributed? We have ourselves no doubt that it is chiefly owing to the neglect of the study of the natural or physiological condition of the functions whose pathological states have occupied almost undivided attention. We cannot hesitate in the belief that if half the acuteness of observation and of the sagacity in the interpretation of phenomena, which have been bestowed upon the subject of inflammation, had been devoted to the establishment of truth in regard to the functions of circulation and nutrition, our pathology would be now possessed of the means of acquiring far more consistency in theory, and more certainty in practice. If we do not hold with Broussais, that inflammation is the foundation of every derangement to which the organized fabric is liable, still it is unquestionably the most important of all elementary forms of disease, constituting a very large proportion of the maladies which affect the human frame, and participating in almost every other.

Those who decry the pursuit of physiology as unprofitable, who think that all the student's attention should be devoted to the study of the morbid conditions of the body, and who imagine that they can rectify the errors of the organic mechanism, as well (if not all the better) in their ignorance of its regular operations, may here be brought to confess the uncertainty of any doctrines founded upon such observation alone, by being required to explain the rationale of the success of two opposite modes of local treatment, possessing the empirical character we have just described. We shall not do our readers the injustice of seriously arguing with them in favour of a position so self-evident as that a good pathology can only be founded on a good physiology; but before entering upon the consideration of the subject which we are to review in this article, we think it desirable to give a brief exposition of the present state of our knowledge in regard to these healthy actions, a peculiar derangement of which constitutes the diseased condition termed INFLAMMATION. We are the more desirous of so doing as we believe that there are few physiological questions respecting which there prevails more either of positive error or vague misapprehension. Without aiming, then, to bring forwards anything of a very original character, we shall endeavour to determine what may be regarded as sufficiently certain and definite to stand as the foundation for pathological generalizations. In so doing we shall only be following, as will presently appear, in the same track with John Hunter.

In the first place, then, we must enquire what are the forces by which the blood is moved, and what are the physical and vital properties of the vessels through which it passes. A great deal of useless speculation on the first of these questions might have been saved, had physiologists brought to the investigation something more than that vague knowledge of physical science which has led some among them, even at no distant period, into most absurd hypotheses. The motor forces assigned by the leading physiologists of the present day may be regarded as originating in, 1, the heart; 2, the arteries; 3, the capillaries. Regarding the first, we need here say but little. No one has any doubt that, in warm-blooded animals, its contraction is the principal agent in the propulsion of the blood to the periphery of the system; and that it operates in a manner strictly mechanical. It is evident, also, that any variations in its operation must influence the whole system alike, except so far as these are antagonized by local peculiarities in the conformation of the blood-vessels. But there is by no means the same certainty that the movement of the blood in the veins is solely due to the *vis a tergo* communicated from the heart, through the capillary system; and still less does it seem probable that this cause can operate where the blood has to be propelled through a double or triple set of capillaries by one impulse. In fishes, for instance, the blood is first transmitted through the respiratory capillaries, for the purpose of aeration; the confluent vessels which collect the blood from these terminate in the general systemic trunk or aorta, in which no pulsation is seen, and by this it is distributed to the systemic capillaries. The blood which returns from the posterior part of the body and from the viscera passes through another set of capillaries, those of the liver and kidneys, before it returns to the heart, the muscular power of which would seem far too weak to overcome such resistance. Indeed, the non-pulsation of the intermediate trunks, the aorta and vena porta, shows that the heart's impulse is not sensibly communicated to them; and the same may be said of the vena porta in birds and mammalia, which, as Hunter has justly observed, should be regarded rather in the light of an artery, strongly resembling, as it does, the aorta of fishes.

As John Hunter was the first who gave due attention to the properties of the arteries, we shall follow him in some detail through his chapter on the vascular system, of which he takes a very comprehensive survey. The following list of the titles of the sections will afford a general view of its contents: 1. General observations on Muscular Contraction and Elasticity. 2. General observations on the elongation of relaxed Muscles. 3. On the structure of Arteries. 4. Of the Vàsa Arteriarum. 5. Of the Heart. 6. General observations on the Blood-vessels. 8. Of the division or branching of Arteries. 9. Of the action of the Arteries, and the Velocity of the Blood's Motion. 10. Of the Veins. These titles will show the comprehensive scope of the contents of this most valuable chapter. That many errors of opinion and some of fact have found their way into it, few will deny; but, notwithstanding all that has since been written on the subject (of most of which Mr. Palmer has supplied us in his notes with an excellent condensed view), no one can be regarded as master of it who has not made himself well acquainted with Hunter's experiments and reasonings. To some of the more important questions here discussed, we shall now direct our reader's attention.

After making some general observations on muscular contraction and elasticity, and on the elongation of relaxed muscles, he proceeds to treat of the structure of the arteries. These he regards as made up of elastic and muscular fibres. The elastic substance is most abundant in the large vessels and in those nearest the heart, gradually diminishing as the distance from this organ increases. From his own observations and experiments, he was led to think that the smaller arteries, or "what have been called capillary vessels," were entirely devoid of an elastic coat, and made up principally of muscular fibres. From the same experiments he also discovered that the larger arteries possessed little muscular power, but that, as they receded from the heart, the muscular power was gradually increased and the elastic diminished. Hence he imagined that there might exist a set of vessels totally devoid of elasticity, although this he conceived to be only in the very extreme parts. He has left no account of the minute and capillary vessels, an omission which, we are sorry, has been but imperfectly supplied in the present edition by a short commentary. Hunter carefully distinguished the elastic from the contractile, or, as he names it, muscular powers of arteries, and has assigned to each their relative importance and use. Elasticity is best adapted "for taking off the immediate force of the heart," and, therefore, predominates in the larger vessels more immediately connected with this organ. The muscular power of an artery, according to him, "renders a smaller force of the heart sufficient for the purposes of circulation; for the heart need only to act with such force as will be sufficient to carry the blood through the larger arteries, and then the muscular power of the arteries takes it up, and, as it were, removes the load of blood while the heart is dilating." Arteries, therefore, afford the most striking instance of an animal substance furnished with two powers existing in the same part, the one to resist mechanical impulses, the other to produce action where action is principally required. Hunter considered that the heart was unable to carry on the circulation without the aid of the arteries; and it was for this reason that the latter, as they decreased in size, were endowed with a greater contractile power. On the question of the agency of the arteries in maintaining the circulation of the blood, we shall extract the following remarks made by Mr. Palmer, which will show the care and ability with which he has executed this portion of his task.

"The arteries were regarded by Hunter as a sort of supplementary heart, scarcely less instrumental in the propulsion of the blood than the heart itself, although it is probable that in this estimate he was as much mistaken on the one hand as Dr. Arnott has lately been on the other, in denying their agency altogether (*Elements of Physics*, 5th edition, p. 358). I shall not, however, enter here into the controverted question respecting the muscularity of the arteries, which appears to me to be a dispute about words rather than facts; for that the arteries possess a power of varying their caliber quite independently of the force *a tergo* is proved by the most indisputable facts. Admitting, therefore, the muscularity of the arteries as a fact, the question arises, in what manner is the circulation affected by it? It is objected to the author's explanation of the action of the arteries, 1st, that if the arteries were extensively elastic, the intermittent stream in the arteries would soon be converted into a continuous one; 2d, that no vermicular or progressive motion in the arteries has yet been observed; 3d, that no diametrical enlargement is visible in the living body; and 4th, that it is impossible to explain the almost perfect instantaneity of the pulse in all parts of the body, on the supposition that the tubes through which it is propagated are in a relaxed state. It is supposed, on the contrary, that the contractile fibres of the vessels operate at the same moment with the heart, so as to induce a rigidity of the whole arterial system, in order that the heart may produce its effect through all the vessels, almost as it would through tubes of metal (*Arnott*, ut supra). Now the first of these objections does not apply, because such extensive elasticity as is supposed to exist does not occur, for even in the dead body the pulse may be imitated; neither does the second objection apply much better, because the very assumption that the pulse depends on the transmission of a wave of blood, presupposes a certain interval of time, and consequently a progressive dilatation. The third objection has been adverted to already, (see note, p. 216;) to which I may add that Poiseuille found, by direct and careful experiment, that the artery apparently dilated with each systole of the heart, that it reacted with a force superior to the impressing impulse, and that the arterial tension was nearly the same in all parts of the system at the same time. The fourth and last objection equally falls to the ground, when it is recollected that no such rigidity, as is supposed to be necessary for the propagation of the pulse, actually occurs, for the isochronism of the pulse is equally conspicuous in all states of the arterial tension, and is scarcely less remarkable in the dead body." (Vol. iii. p. 229.)

There is little doubt, we think, that Hunter was right in ascribing to arteries a property somewhat analogous to that of muscular irritability, and recent enquiries into their intimate structure would seem to justify this doctrine. It is true that the authority of Berzelius should lead us to regard their middle coat as destitute of fibrine, which has usually been considered as the characteristic ingredient of muscular fibre: but we fully agree with Dr. Allen Thomson in the opinion that "although we must admit the importance of such observations, as establishing chemical distinction between muscular substance and the texture of the middle coat of the arteries, they do not warrant the conclusion too hastily deduced from them by some, that this coat cannot be irritable, or does not possess any of the same properties as muscle, the existence or non-existence of which must be ascertained principally by physiological evidence."* Although the structural difference has, by most observers, been regarded as equally decisive, it would appear to have been so on insufficient grounds. The comparison has been made with the fasciculi of the voluntary muscles, in which *fibres* are seen made up of *fibrillæ*, inclosed in a sort of sheath, and bound together by annular or spiral bands, which have given the appearance of striæ. In the muscular fibre of the alimentary tube, however, as in that of the lower animals, the fibrillæ are separate, unmarked by striæ, and irregularly interlaced instead of lying parallel; and in all these respects they closely correspond with the fibrous coat of the arteries.† Between these tissues, again, there is more physiological resemblance than either possesses to the fibre which composes the voluntary muscles. Both of the former are acted on by direct stimuli, and not at all, or at least very imperfectly, through their nerves; whilst the contraction of the latter excited by direct stimuli is very trifling, and can only be effectually induced by an irritation transmitted through the nerves. We shall not detail the experiments which prove that a property of this kind exists in arterial tissue; they are to our minds quite satisfactory; but the question then arises, what influence it has in causing or modifying the circulation of blood through the vessels. It is universally conceded that to their *elasticity* only is due the gradual equalization of its flow, and the diminution of the pulse-wave, as we proceed from the centre towards the circumference. We do not think that there is evidence of any action like that of the pulsating

* Cyclop. of Anat. i. p. 665.
† Skey in Phil. Trans. 1837; and Mandl, Anatomie Microscopique.

dorsal vessel of insects, in which a kind of peristaltic or vermicular contraction propels the blood from behind forwards,—the vessel being, in fact, a series of hearts, one for each segment, which come into play consecutively. Still it does not appear that mechanical distention stimulates the vessels to contract again with more than the force which dilated them; and this, acting through the whole arterial system at once, may, as Hunter supposed, be an important auxiliary to the propelling power of the heart.

But we cannot agree with many physiologists in attributing to this secondary influence all that is required to make up for the deficiency in the heart's action. A more important source of movement, and one which has been very generally overlooked or undervalued, originates in the capillary system; but before enlarging upon this, we must advert to what we conceive to be the principal office of the muscular coat (for so we feel justified in terming it) of the arteries, and especially of the smaller branches where it is most developed. We fully accord with Mr. James* (to whose treatise Dr. Macartney seems to us to be under more obligation than he has acknowledged,) in thinking that the elastic coat of the arteries enables them to "enlarge or diminish their caliber, to *regulate* the quantity of blood conducted through them to any part or the whole, according to the demand that may be made, under circumstances constantly varying." This appears to be Dr. Macartney's opinion, as far as we can gather from his phraseology, which sometimes exhibits an almost Hunterian obscurity. "The small arteries never yield to mechanic force, but always change their character from a new disposition, arising out of a new state of feeling." (p. 126.)† Every physiologist knows that arterial trunks vary much in diameter under different natural conditions of the organs which they supply; and this difference will be found to accord with variations in the activity of the processes of nutrition, secretion, &c. that are characteristic of the capillaries into which they respectively divide. The idea that the dilatation of arteries is ever occasioned by distention *a tergo* (except under very peculiar circumstances), or that their contraction is due to the withdrawal of internal pressure, has recently been ably controverted by Dr. Graves,‡ to whose valuable papers on the subject we would refer such of our readers as wish to see the question treated at large.

We shall advance, however, a step further, (in which we believe that we are again in accordance with Dr. Macartney, although he does not state the doctrine very explicitly,) and maintain that this alteration in the caliber of the arteries, in proportion to the amount of blood which they have to convey, is governed by the ganglionic nerves distributed so universally upon their tubes. We infer this from the analogy in structure and function between the elastic coats of the arteries and the muscular tunic of the alimentary canal. The vermicular motion of the latter is unquestionably influenced by mental emotions, and by sympathy with other processes of the system, and these influences can scarcely be

* Observations on Inflammation, (p. 9), 1832.

† We shall take occasion further on to discuss the propriety of Dr. M.'s use of the terms *feeling*, *sensibility*, &c. to imply changes in which *consciousness* does not participate.

‡ Medical Gazette, No. 553, &c.

conveyed in any other way than through the ganglionic nerves that supply them, whose function appears to be to connect the purely *animal* with the *organic* functions. Just so we conceive it to be with the arteries. That they are capable, in some parts of the body at least, of being excited to special changes through the nervous system can scarcely be doubted when we consider the phenomena of blushing and some cases of *erection*, in which sudden and spontaneous dilatation of the smaller arteries is known to occur; and the facts which we shall hereafter adduce leave small ground for hesitation, as to their being sympathetically influenced through the same channel in diseased as well as healthy conditions.

We alluded in our last Volume (p. 181) to the general arguments which, in our opinion, substantiate the doctrine that the changes which the vital fluid undergoes in its passage through the capillaries, whether these changes be of a nutritive or a secretory character, have an important influence on its movement through them. Several of these are well stated by Mr. Palmer in his Summary of this part of Hunter's Treatise (p. 231-2); and to this we may refer our readers, premising, however, that we do not understand them as proving any *mechanical* action of these vessels, which appear as inert tubes under the microscope,—but rather as leading to the belief that a new set of *attractions and repulsions* (whether of a physical or vital character) are created between the particles of the blood and the walls of the passages through which they move, by the action which, in the normal condition, takes place between them. "The inference which may fairly be drawn from the whole of these facts," concludes Mr. P., "seems to be that the capillaries possess a *distributive* power over the blood, so as at least to regulate the local circulation independently of the central organ, in obedience to the necessities of each part." We deem ourselves further justified in laying down as a general proposition that—*the circulation of blood through the capillary vessels of any part will have a rapidity proportional to the activity of the normal changes which the blood undergoes in them; that it will be accelerated if, from any cause, these processes take place with unusual energy* (*in which case the vitality of the part may be regarded as exalted*); *that it will be retarded if these processes are in a depressed condition; and that it may be entirely checked by their total suspension.*

It is not a little curious that those physiologists who deny that the capillaries have any kind of influence on the motion of the blood through them, are driven, by the force of the facts to which we have alluded, to make certain exceptions, in which nearly all is conceded that is demanded by their opponents. Thus, we find Müller stating that the movement of the blood is entirely dependent on the action of the heart, and presently afterwards alleging the existence of an attraction or affinity between the blood and the solids which it supplies, in the "turgescence, turgor vitalis, or orgasm," which is observed to take place in certain parts of the body, independently of the heart. He cannot conceive how such an attraction can become one of the regular moving forces, however, since it would cause congestion, by rendering the blood stationary in the capillaries, "unless," he adds, "it be again admitted that this attraction of the capillaries for the blood is exerted only while the blood retains its arterial character, and ceases when it has become venous." Now, that this hypothesis is the true one, is, we think, supported by a pretty strong body of evidence. It is well known that, when there is any local excitement to the processes of nutrition or secretion, an increased flow of blood towards the part speedily takes place; and not only this, but an increased circulation of blood *through* the part occurs, quite independently of any altered action in the heart. This we find to be the case at puberty, as to the whole generative system; during pregnancy, with regard to the uterus; during lactation, in the mammary glands; probably with regard to the brain, also, when its functions are unusually, but normally, excited; and during the formation of new parts in the embryo. In vegetables, also, it is acknowledged that an afflux of fluids takes place towards parts in which active nutritive processes are being performed, even though the latter be artificially stimulated at an unusual time; and if the superfluous portion of these fluids were not returned or exhaled, the tissues would soon exhibit the unhealthy dropsical appearance which they assume when exhalation is checked by want of light.

We find the converse of this action occurring where there is any diminution or obstruction to these processes. If the diminution of the circulation through any part be one of the changes natural to the economy, we find that congestion is prevented by a progressive contraction in the arteries supplying it, as in the case of the uterus after parturition. But if the processes of secretion or nutrition be interrupted by extraneous causes, congestion is the immediate result; for the capillary circulation is *thereby* rendered inactive, and the blood accumulates in the affected vessels. We cannot have a more striking example of such an occurrence than in asphyxia, the true pathology of which has a most important bearing on this and other important physiological questions.

We have dwelt much upon the question of the independent action of the capillaries, because it is one of peculiar practical importance, especially in regard to the proximate cause of congestion and inflammation. It affords us at once the key to the comprehension of a great majority of cases of local disease, in which the former is a leading symptom. We do not mean to say that congestion of the different vessels is in *all* instances the *result* of inactivity in the nutritive or secretory processes in the part affected; but we are persuaded that it will be found to be so in a great majority of cases. If so, the obvious curative indication is to excite the capillary circulation by stimulating these processes. How successful such a plan is where we have the means of adopting it, we need hardly point out in detail. It will be sufficient to allude to the relief immediately effected by artificial respiration in cases of congestion of the pulmonary arteries from obstruction to the access of air; and to the influence of mercury in reducing the congested state of the liver which is so often found to accompany a deficient secretion of bile. The deficient secretion is often regarded as the *result* of the congestion; but the analogy of the lungs is, we think, quite satisfactory upon that point.

The doctrine which we have been upholding, in regard to the influence of the capillary changes in the blood on its circulation, serves also to explain the mode in which the action of the ganglionic nerves affects this function. There can be no doubt that the nutritive processes are regulated and harmonized by the operation of this system; and that the *organic sympathies* are chiefly communicated through it. It is easy to see, therefore, that, by acting like other stimulants or sedatives on the nutritive and secretory functions, innervation may greatly affect the motion of the blood; but that it has any other channel of influence has never been proved. Sudden and violent impressions on the nervous centres, such as powerful electric shocks, extensive mechanical destruction of nervous tissue, especially by crushing, and certain sedative agents, are found to arrest the capillary circulation almost instantaneously through every part of the body. These act, it is well known, upon the general vitality of the system, and seem to destroy that of the blood, as well as of the solids; so that there is a double reason for the remarkable effect in question. And local destruction of vitality by spontaneous gangrene has been found to check the flow of blood towards the parts, although it was proved, by subsequent examination, that no mechanical impediment existed. For ourselves, we are unable to perceive a single valid objection against the doctrine we have been supporting. It harmonizes completely with all the phenomena which a careful comparison of organized structures of different kinds brings to bear upon it, as well as with those supplied by pathological observations. It may be objected to it that it is deficient in precision, and that the idea of the reaction between the blood and the surrounding solids having anything to do in the propulsion of that fluid is a mere hypothesis; but we would reply, that we know just as much of the mode in which it operates as we do of the excitement of muscular contractility by the application of a stimulus; and that the action of the heart itself is quite as inexplicable as that which we have ascribed to the capillaries.

We shall now request our readers to follow us in another branch of the enquiry—that which relates to the vital properties of the blood; and here we shall again take Hunter for our guide, prefacing the discussion, however, with a few remarks on his treatise, entitled "General Principles of the Blood." This gives a comprehensive view of the mass of the blood, as composed of different parts,—of coagulation and its effects,—the serum,—the red globules,—the quantity of the blood, and course of its circulation,—the living principle of the blood,—and some unconnected experiments respecting this fluid. At the time Mr. Hunter lived, animal chemistry was in such a rude and imperfect state, that he was little disposed to place reliance on the aid which it afforded, and hence he endeavoured principally to ascertain the properties of the blood from other sources. Much additional light has been shed on all these points over which chemistry presides; so that, while Hunter may be said to have minutely and accurately sketched the natural history of the blood, none of the results furnished by modern analysis appear in the original pages. This, however, has been amply remedied in the present edition by the excellent notes which are appended, and which not merely correct the errors into which the author was betrayed, but, by setting before the reader, in a plain and lucid manner, the more recent chemical discoveries, carry down the subject to the knowledge of the present day. It is only, indeed, within the last few years, comparatively speaking, that many of the most interesting questions regarding the blood have been brought forward and discussed with vigour; we allude to the presence of peculiar substances in the mass, or in the secretions immediately formed from it, the changes which the former is observed to undergo under various circumstances, and the relation which these bear as to cause and effect in the phenomena of disease. Of all these things Hunter was nearly ignorant; for, although the humoral pathology had been known for centuries, it had, during his days, sunk before the more specious and tangible system of the solidists. It is doubtful what share he would have taken in this discussion, as there is almost nothing in his writings to lead us to form an accurate conclusion.

The point, above all, for which Hunter was distinguished, and on which he separated himself from most other physiologists of his time, was his doctrine of the *life of the blood.* It ought to be remembered, however, that although he was the warm advocate of this theory, he was not, by any means, its original projector: we find the same idea entertained by Harvey, who considered the blood as the *primum vivens* and *ultimum moriens* in every animal; and it is not improbable that he caught the hint from some less definite expressions made by Aristotle. We may trace it still further back in the writings of the inspired lawgiver of the Jews: "Only be sure that thou eat not the blood; *for the blood is the life*, and thou mayest not eat the life with the flesh."* The notion of attaching vitality to a fluid appears, at first view, not a little extraordinary, and accordingly drew down upon Hunter severe ridicule from his opponents.

> "To conceive that blood is endowed with life while circulating is perhaps carrying the imagination as far as it well can go; but the difficulty arises solely from its being a fluid, the mind not being accustomed to the idea of a living fluid. . . . . When all the circumstances attending this fluid are fully considered, the idea that it has life within itself may not appear so difficult to comprehend; and indeed, when once conceived, I do not see how it is possible we should think it to be otherwise, when we consider that every part is formed from the blood, that we grow out of it, and if it has not life previous to this operation, it must then acquire it in the act of forming; for we all give our assent to the existence of life in the parts when once formed. Our ideas of life have been so much connected with organic bodies, and principally those endowed with visible action, that it requires a new bend to the mind to make it conceive that these circumstances are not inseparable." (*Palmer's Hunter*, iii. p. 104-5.)

Hunter asserted that the blood was not only alive in itself, but the support of life in every part of the body; for if it had not this living principle, it would be, in respect of the body, an extraneous substance. It is not alleged, indeed, by any of its supporters, that this doctrine is capable of being demonstrated: but only that it is impossible for us otherwise to explain many of the changes which the blood undergoes. We have not space, nor is it worth while, to follow Hunter through his speculative argument; for, although sufficient evidence may be adduced to show that blood is affected by circumstances which have no influence on other fluids, nothing like *proof* of its possessing a distinct and independent vitality of its own was brought forward by him. "The argument for the vitality of the blood," as Mr. Palmer justly observes, at the end of a very judicious summary which he gives of Hunter's main facts and reasonings on the subject, "is strictly a cumulative one, and must be viewed in its totality, in order to estimate its real force. Taken

* Deut. xii., 23.

altogether, the preceding facts raise a strong presumption in its favour, not to say a high probability." The two main points on which the doctrine with Hunter rests, are the circumstances affecting the coagulation of the blood, and the phenomena attending union by the first intention. The former he considered one of the greatest proofs that this fluid possesses life, for he conceived coagulation to be "an operation of life." Few physiologists, either British or continental, continue, we believe, to hold this part of Hunter's doctrine; but we cannot help thinking that it has been abandoned upon very slight cause. The evidence in its favour, involving many considerations which did not occur to Hunter, has recently been most ably summed up by Dr. Alison;* and we do not think it necessary, therefore, to enter into any detail on the subject at present. We may observe, however, on the nature of the facts brought in its support, that they are such as show an analogy between the effects of various agents on this process and those of the same on operations confessedly vital, which, in both cases, frequently differ from what might be supposed to be their physical influence. If the fibrine of the blood be regarded as possessed of vitality under any circumstances, we cannot see any difficulty in supposing its properties to be such that it shall remain fluid when in movement and in contact with the coats of living vessels, and that it shall concrete into a solid when withdrawn from these influences; this concretion being still, however, a result of the properties with which it became endowed when its elements were united in its peculiar proportions.

Those who deny vitality to the blood on account of its fluidity, or, in other words, maintain that its properties are only those of inorganic matter, seem to forget that other fluids unquestionably exhibit vital properties. "We must not believe," it has been philosophically remarked by Andral, "that the manifestation of life only occurs where there is an organization such as we find it in the higher orders of animals, such as we are therefore in the habit of representing it in our own minds, and such as we conceive it to be in all cases." The proportion of fluids to solids in many of the inferior organisms is much less than in the blood. A large *medusa*, for example, which, when taken out of the water, weighs fifty ounces, is reduced, by drying, to a few grains; and the same might be said of some of the aquatic cellular plants; but it may be objected that these possess a definite organized structure. What, then, will be said of the semen of animals, or of the fovilla of the pollen of plants, which so nearly resemble the blood in character? Will it be maintained that any mixture of chemical products can imitate the effects of these? Or, to take a still more analogous case, the semi-fluid granular mass which unites to form the germinal membrane of the ovum; the changes which this undergoes are so completely analogous to those of the blood in process of organization, that they are evidently governed by the same laws; and will any one assert that these are laws of physics rather than of vitality? By such comparisons, it appears to us, we may not only refute the objection urged against the doctrine of the vitality of the blood, on the score of its fluidity, but obtain an affirmative argument of no mean value. To the series of proofs derived from the changes which

* Supplement to Outlines of Physiology, pp. 31-3.

the blood undergoes whilst still in its vessels, or extravasated among living tissues, we shall presently recur,—concluding this part of the subject with the following judicious remarks, which we extract from Dr. G. Burrows' Croonian Lectures on the Pathology of the Blood:* "It is perfectly true that, if the blood be regarded as a mass moving through the larger vessels, and be compared with any organized tissue, or with the parenchyma of any organ, there is no doubt a great difference between the physical qualities of the living fluid and solid. This dissimilarity, however, no longer exists, when a comparison is made between the blood in the capillaries of an organ and the surrounding structure which it nourishes and forms; in this part of each organ the blood and the tissue are intimately blended together; [as intimately, we may add, as the solids and fluids in the tissues of the acalephæ, in which little circulation takes place.] In every structure there is a point where the blood must be regarded as becoming extravasated—where it mingles and combines with and renovates the surrounding tissue—where, in fact, it becomes organized, and where its vitality is no longer a matter of doubt."

The strongest evidence that the coagulation of the blood, when drawn from the living body, is the result of the vital properties which it still retains, appears to us to be derived from the fact that a process essentially the same is the preliminary to the organization of the blood within its natural cavities. It is scarcely to be believed that, if this fluid be at any time possessed of vitality, its *death* should be the connecting link between its liquid and its evidently organized condition. That a similar coagulation occurs in plastic lymph, as a preliminary to its organization, has long been known; but this coagulation has been regarded by many as of a different character from that of the blood, from its being supposed that the red globules played an important part in the latter, and that coagulable lymph was a product of inflammation, essentially different from anything which previously existed in the body. Now, however, it is known that coagulable lymph and the liquor sanguinis are almost identical, and that the red particles are passive, or nearly so, in the coagulation of the blood. There is, therefore, a strong argument from analogy for considering this change in the blood in the same light as in the lymph. But, it may be argued, we do not find organization following it in the blood, as in plastic lymph, it being now generally allowed that blood effused into wounds or cavities is never converted into new tissue. Recent observations have shown, however, that, under certain conditions, blood *is* organized after coagulation in its natural cavities; and this process appears due to the influence of the vital properties of the tissues with which it is in contact. We shall not, at present, adduce the proofs of this position, but shall be satisfied with referring again to the very valuable Croonian Lectures of Dr. Burrows, where our readers will find them well displayed, and with extracting his summary of facts relating to the changes of a vital character which the blood undergoes in the vessels. "Thus we have instances, both in the heart and arteries, of the coagulation of the blood during life; of the various changes taking place in that coagulum, from a state of simple, unorganized, discoloured

* Med. Gazette, vol. xviii.

fibrine to an organized solid, often eliminating within itself fluids found in other organized parts, such as serum or pus; at other times the fibrine is converted into cartilaginous or osseous structure, or into matter resembling medullary sarcoma, which may or may not coexist in other organs of the body." The same changes are found to take place in blood stagnated in the veins; they result from any circumstances which impede the circulation, either by depressing its moving powers or by mechanical obstruction; and they are also produced by inflammation of the coats of the veins, which occasions the coagulation of the blood stagnated in them. The kind of structure into which the coagulum is ultimately converted appears to be much influenced by the nature of that with which it is in contact. Thus Andral mentions a case in which an osseous cyst, which had apparently originated in a fibrinous clot, was found beneath the valves of the pulmonary artery; and a large quantity of osseous matter was deposited in the lining and walls of the right ventricle. Such facts were noticed by Hunter, who embodied them in a general law; that when the blood has once coagulated in the living body, and become a solid, it "then changes into this or that particular kind of substance, according to the stimulus of surrounding parts."

It is remarkable that after the doctrine of Hunter, as to the organizability of coagula of blood effused into the tissues, had been abandoned by most succeeding physiologists and pathologists, it should be again revived with such additional evidence as to demand our reception of it as a fact. Such appears to us the case; for the late investigations of Andral, Carswell, and others leave no doubt that small coagula of blood thus effused may undergo most if not all of those changes which have been just stated to occur in blood coagulating within its own vessels; acquiring a distinct structure, and forming adhesions to the surrounding parts, and occasionally undergoing various morbid transformations. It is evident that our elementary notions of disease must be very erroneous if facts of this kind are left out of view; and we have given this brief sketch of the present state of our knowledge regarding the vital properties of the blood, because we deem with Hunter that on no other foundation can we erect any true theory of inflammation.

Before proceeding to enquire what inflammation *is*, we shall find it useful to determine what it *is not*. There are two conditions of the vascular apparatus of any organ which are liable, on a superficial view, to be mistaken for it; and these, although frequently confounded, are as different from one another as from the state with which we would contrast them. These are *determination of blood* (as it is commonly but erroneously called) and *congestion*. In the first there is an increased afflux of blood to the organ, resulting from increased activity in the vital processes to which it there ministers; and this increased activity may be either a part of the regular series of operations performed by the organism (as in the enlargement of the uterus, mammary gland, &c.); or it may be of an abnormal character, in which case it is generally accompanied by deficient action in other parts. How common is it to meet with this activity of function in the brain, accompanied by flushing of the face, throbbing of the carotid and temporal arteries, and the other symptoms which are popularly known to attend "determination of blood to the head," whilst the extremities are liable to coldness and lividity, marking torpor of the circulation in them? Now, it is sufficiently evident that the amount of blood at any time contained in an organ thus excited, whether normally or the contrary, will be greater than in the same whilst performing its regular actions only; yet this state is not one either of congestion or inflammation. An increased quantity of blood is attracted to it, but there is no stagnation of it in the vessels; no action of an abnormal *character* takes place between the fluid and the tissues which it supplies, the difference from the natural actions being only in *degree;* and the blood, propelled through the capillaries with an accelerated motion, is freely returned by the venous system. Although this condition *is not* inflammation, we shall presently see that it may border very closely upon the first stage of that morbid action; and every one knows that organs which are most liable to such states of excitement are also most prone to take on the inflammatory process.

In *Congestion* (using this term, however, in a more restricted sense than some writers seem to intend,) there is an absolute retardation of the flow of blood through the organ, together with an accumulation, either in its afferent vessels alone, or in both systems, according to circumstances. This retardation and accumulation may result from several causes. A more frequent *cause* of it than is usually suspected is, we are confident, a depression of the vitality of the organ, and a consequent inactivity of those processes which are essential to the maintenance of the rapidity of the circulation through it. We find an excellent exemplification in the state of asphyxia, in which the suspension of one of the conditions of those processes, the access of oxygen, causes congestion of the afferent vessels, from the accumulation in them of blood which is not able to pass through their capillaries. And we are certain that this principle may be more generally applied than it is in the diagnosis and treatment of disease, with very great advantage; for when the inertness of the nutritive and secretory processes is regarded as the *cause* rather than the *effect* of the congestion, our treatment will be applied rather to stimulate them than to relieve the congestion by the abstraction of blood. Now we shall hereafter find that this kind of congestion bears nearly the same relation with the second stage of inflammation that determination of blood does to the first; the principal difference being, as in that instance, that in inflammation there is a change in the *character* as well as in the *degree* of the natural actions. Here again, however, we find the two states often approximating very closely; since a morbific cause is peculiarly liable to operate in producing inflammation on an organ whose vital actions are already disordered. Of the other causes of congestion, arising principally from mechanical obstruction to the passage of the blood through the vessels, owing to its stagnation in other organs, compression of the vessels, &c., we shall not here dwell, since they have little to do with the theory of inflammation; we shall revert to them, however, when following Dr. Carswell through his account of the sensible changes produced in the tissues by the two conditions respectively.

The chapter in Hunter's Treatise devoted to the Fundamental Principles of Inflammation is, without any exception, the most laboured and intricate in the whole volume; and we shall, therefore, not attempt to give any analysis of it. But it is, at the same time, replete with interesting matter, and contains the rich outpourings of a vast and enlightened mind; a fountain of knowledge at which the pathologist may drink deeply. What a world of thought sometimes lies in a single sentence? We can only notice, in passing, the division appropriated to the consideration of the progress of inflammation in different structures, and the influence which structures have in modifying its character. Here has Hunter developed, in his own masterly style, the changes to which inflammatory action gives rise in the different tissues, and which have since his day occupied so conspicuous a place in the writings of succeeding pathologists; and here, also, may be distinctly found the germs of those brilliant views afterwards brought forth by Bichat, but without any acknowledgment of the source from which they were originally derived.

Hunter considered that inflammation was only a disturbed state of parts which required a new but salutary mode of action to restore them to that state wherein a natural mode of action is alone necessary; and that, from such a view of the subject, inflammation was not to be reckoned a disease, but as a salutary operation consequent either to some violence or some disease. We need scarcely stop to remark that such a notion is, on the very face of it, far too exclusive to be correct. Every deviation from the natural state cannot be regarded as salutary; and although inflammation is no doubt set up by nature in certain cases to effect a certain end, it is, at other times, the most dangerous and fatal disease which could arise. We almost wonder how Hunter could have been betrayed into such an error; and, indeed, we find him, with that disregard of minute consistency which characterizes minds of his ardent and enquiring character, acknowledging it in the very same page. It is not, however, in this chapter, but in that which comprehends Adhesive Inflammation, that Hunter discusses the state of the vessels in inflammation, the local and constitutional signs of the disorder, the effects in the system according to the structure of the part affected, and the remedies necessary for its removal. One of the most interesting points of enquiry, and which has received a large share of attention, and been made a subject of keen discussion, is the actual state of the vessels of an inflamed part. Considering the amount of labour and time spent in this investigation, we are naturally curious to know, even at this distant period, in what conclusions such an acute observer as Hunter rested. It does not appear then, from anything that we can detect, that he had ever examined the process of inflammation experimentally, or as it is seen going forwards in the transparent parts of an animal texture, and he seems to have rested content with theorising on the subject. From this it followed that, while some of his notions were correct, he was at the same time drawn into inconsistent statements, evidently experiencing the greatest difficulty in making up his mind on this abstruse question. No information can be drawn from such reasoning as he offers, founded as it was upon data so insufficient; for wherever speculation usurps the place of experiment and observation, we only hunt after the shadow and lose the substance.

Only one point was prominently insisted on by Hunter—the increased size of the vessels, coupled with increased power of action; but he does not define what he implies by the latter: "It must appear," he says, "that a much larger quantity of blood passes through parts when inflamed than when in a natural state, which is according to the common rules of the animal economy; for whenever a part has more to do than simply to support itself, the blood is there collected in larger quantity . . . . What the action is, or in what it differs from the common action of the vessels, is not easily ascertained." He seems to have had some difficulty in reconciling increased action of the capillaries, which, it might be supposed, would produce their contraction, with the fact of their dilatation. He gives a theoretical explanation of this discrepancy, and endeavours to establish his doctrine of increased action by reference to a great variety of phenomena, many of which, however, have no more than an analogical relation with it, and many more may be explained on other principles. Of these Mr. Palmer has given an excellent condensed view in his note to the chapter in question.

But although we differ from Hunter so completely as to the nature of the change of action in the capillary vessels, we cannot help thinking that, in referring the proximate cause of inflammation to that change of action, and in considering the modification in the size of the vessels and in the rapidity of the circulation of the blood through them as secondary phenomena, he was approaching the true point of investigation much more nearly than some of his successors, who have considered these last changes (which, as we shall presently see, are but effects of others,) in the light of proximate causes. "Inflammation," says Mr. Palmer, in the spirit of Hunter, "is an act which involves all the functions of a part." The blood circulating through it, as well as its own tissues, are altered in structure and properties. "More or fewer of these changes invariably attend inflammation, which cannot therefore be supposed to consist in a simple alteration of the circulation, but principally of those vital affinities subsisting between the vessels and their contents, which are the peculiar attributes of life." (p. 328.)

The theory of inflammation propounded by Dr. Macartney appears to us even more unsatisfactory than that of Hunter. Neglecting all consideration of the blood, and of the vital processes to which its motion through the capillaries is at once subservient and in part due, he has looked for the proximate cause of this condition only in the state of the vessels, which we cannot but regard as either a *result* of the primary alteration, or, at most, only a *collateral* change. It is but fair, however, that he should speak for himself, and we shall therefore give his opinions in his own words. In the first place, speaking of the *modus operandi* of the local causes of inflammation, he says:

"It is obvious that they all act by making such impressions on the sensibility of parts as dispose the arteries to assume the inflammatory state . . . . . That even local injury excites inflammation, by operating on the sensibility, is proved, by its effect being proportioned to all the circumstances connected with organic sensibility, and not measured by the degree of mechanic destruction. As one illustrative example we may mention that a small punctured wound of the tendinous parts of the hand or foot would naturally induce more inflammation than blowing off the whole hand or foot by an explosion of gunpowder. The most striking evidence we have of the impression on the sensibility being the sole cause of inflammation, is found in the fact of all the naturally close cavities inflaming from being opened, and thence placed in a *new state of sensation* to them . . . . . It is the organic consciousness or sense of exposure, or rather of imperfection, which causes inflammation in cavities that are naturally close." (pp. 111-2.)

This passage will afford us the key to Dr. Macartney's very peculiar phraseology, upon which we must take leave to make some strictures. It is entirely based on the doctrines of Bichat, which have been abandoned, in this department at least, by all the most intelligent physiologists of the present day. We refer particularly to that of "organic sensibility" being dependent upon the ganglionic system of nerves, which is to it what the cerebro-spinal system is to the animal sensibility. We shall hereafter see that the whole of Dr. M.'s theory of inflammation is erected upon the hypotheses (for such we must consider it), that the "organic sensibility" of the nerves of the part is first acted on; and that is the cause of all the other changes. In accordance with this he is led to the use of terms which we cannot but regard as worse than useless; since they not only complicate what is in itself sufficiently simple, but lead the passive recipient of them to imagine that something is explained, when in reality it is only involved more deeply. What is gained, we would ask, by attributing the contraction of the bladder on its contents (so that, whatever amount of fluid it may hold, it is always full,) to the "organic consciousness of vacuity?" (p. 112): or the disposition in the arteries to contract when blood is withdrawn to the "sense of danger felt" in them? Truly, in regard to the intelligibility of such expressions, we think that they are far below the celebrated "stimulus of necessity" of John Hunter. The latter had the advantage of not appearing to account for what seemed unaccountable; but plainly confessing the author's ignorance of the cause as any other than a law of the economy; whilst Dr. M.'s explanations, which appear to us about as valuable as those of the physiologists who regard everything as *vital* which they cannot explain in other ways, have the same injurious effect in tending to check enquiry, by holding up that as accounted for which is in reality only expressed in different and, to our minds, most objectionable language. It seems to us an abandonment of all precision to use the terms *sensibility* and, still more, *consciousness* in any but a psychical import. The more abstract language we employ, the more necessary it is that we should keep within the bounds of rigorous definition. And we are quite sure that the metaphysics of physiology will never assume a truly scientific form until its language is much more precise than at present. Most French physiologists of the present day have abandoned the term "sensibility" as applied to the simple vital properties of any part, which is all that it can thus express; and though some use the term *sensation* in the same import that in Britain is attached to *impression*, that is, a change in the condition of the afferent nerves, which, propagated to the sensorium, produces consciousness of the presence of an external object, it has only reference to the cerebro-spinal system. If, then, we regard this as a retrograde movement on the part of Dr. Macartney, still more must be reprehended his use of the term *consciousness* to imply a state in which *the mind* is not concerned. To do so is to break down all barriers between physics and metaphysics, the corporeal and the psychical. Even his prototype, Bichat, did not venture so far as this, but reserved the term to imply a state of *mind* in which the individual, *le moi*, is (we cannot phrase it in any other way) *conscious* of his existence. And will Dr. M. tell us that this condition can exist in the ganglionic system or the organs connected with it? It is very true that he qualifies it by the prefix "organic;" but why use it at all, since nothing is gained by it? We venture to prophesy that should Dr. M.'s opinions be made the subject of controversy a hundred years hence, they will stand a fair chance of being as much misinterpreted as those of Whytt have been in recent times; Whytt's "soul," as the originator of the "vital and involuntary motions," being precisely the analogue of Dr. M.'s "consciousness." We can imagine some learned critic of the next century following the same track, in the exposition of his opinions, as we have lately trodden, in the enquiry after those of Whytt; and toilsomely seeking, in the writings of the almost forgotten Bichat, and, peradventure, in our pages, for the solution of the mystery.

We must now return from this digression to criticise, not Dr. Macartney's language, on which we have sufficiently commented, but his doctrines. The first part of his chapter, on the Proximate Cause of Inflammation, is devoted to an enquiry into the structure and properties of the arterial tubes; and as his opinions on this subject lie at the foundation of his explanation, we shall quote such portions of his exposition as will serve to convey a fair idea of them, referring to the work itself for the arguments in their behalf.

"It is admitted by all physiologists, that in proportion as the elastic coat of the arteries declines, these vessels possess the power of enlarging and contracting the size of their tubes. . . . . That there is such a thing as a positive and active extension and dilatation in the arteries, and other tissues similarly endowed, I think, cannot be denied, since we have evidence of dilatation taking place, under circumstances, and to an extent, that could not be produced by a mere cessation of the contractile action; and, as we see in all cases where vessels are dilated, or surfaces are extended without mechanic force, the causes are some excitement or irritation, or a necessity for the performance of some new or more vigorous functions. We have, therefore, the right to assume, that the dilatation or extension of parts is their most active condition, and that their contraction is the negative or passive state, it being that into which tissues fall when they are free from stimulation, or when their functions cease, or when they are deprived of nervous influence, or when the causes which produced dilatation are withdrawn. . . . . In arteries this *tonic action*, if not limited to the inner tunic, is most remarkable in those branches which are composed only of this tunic and common cellular substance. Arteries are observed to exhibit three states: 1st, the *middle* or *ordinary* one, which is consistent with natural circulation, growth, and secretion; 2d, the *excited state*, in which they are dilated, and thereby admit an unusual quantity of blood; and 3d, the *passive state*, in which they receive a smaller quantity of blood than usual, and sometimes none at all." (pp. 116-8.)

In these statements there are several controvertible points. In the first place it has been shown, by the recent observations of Schwann, that the capillaries have a fibrous coat at least as well developed, in proportion to their caliber, as that of the larger arteries. Dr. M. is, therefore, in error in stating that the tonic power of arteries increases with the decline of their elastic tunic; and we maintain, on the other hand, that all their changes in diameter, as well in the capillaries as in the larger trunks, are under its control. But further, we deny *in toto* Dr. M.'s position that a dilated condition of these tubes is an evidence of the activity of their functions. In the facts which he adduces in support of the doctrine, he appears to us to manifest a strange confusion of ideas. Thus he brings forward the dilatation of the arterial trunks, and the thickening of their coats, in cases of determination of blood to particular parts, as an evidence that the dilatation of the capillaries is an active condition. The two phenomena are essentially distinct. The dilatation of the arterial trunks, occurring wherever there is an increased demand for blood, has only reference to their function as canals for its conveyance; and it would be very possible to attribute it solely to the increased nutrition of their coats which takes place under such circumstances. The dilatation of the capillaries, on the other hand, has reference to their functions as nutrient and secreting vessels; and it by no means follows that *these* functions are more active when their caliber is increased, so as to allow a larger stream of blood to pass through them. In fact, we shall find that the exact contrary is the case. The same objection will apply to all the other arguments which Dr. M. has adduced in proof of his position. We by no means deny that, what he terms *vital dilatation*, that is, the enlargement of the caliber of a canal, independent of mechanic force, does take place in the living body; but we deny that it is always to be regarded as the result of a state of increased activity in the tissues concerned, and more especially in the instance of the capillary vessels. We shall now follow Dr. Macartney to his explanation on the *proximate cause* of inflammation, forewarning our readers to make due allowance for his peculiar phraseology.

"When an injury has been committed on any part of the body, the natural sensibility of that part is roused to a consciousness of what has been suffered, either instantaneously or in a very short time. This local feeling or impression, or whatever it may be called, is distinct from the sensible pain of a wound, and may exist in the greatest degree where little or perhaps no pain is felt. At other times it may be coincident with common pain, although when perceived it is distinguishable from it. In general the organic consciousness is distinct from that of the individual, unless the sense of injury be communicated to the whole nervous system, and then the person feels a degree of agitation and alarm which he cannot understand or account for. On some occasions we observe the immediate effect of the impression shown by the vessels of the part, as when the circular blush around the puncture at the moment of vaccination, gives intimation of the sensibility of the part, to the admission of an animal poison; and sometimes the individual is conscious of a peculiarity of sensation from the contact of other morbid poisons, when the organization has not been injured, but has merely received the impression. As we have sufficient grounds for assuming that an organic sense of injury or danger, modified according to the causes which produce it, necessarily precedes inflammation, it is reasonable to conclude, that this is the proximate or essential cause of inflammation, and the opinion seems to be confirmed by the history already given of the constitutional and local causes; as also by the contemplation of the kind of sensibility and vital properties which belong to the tonic tissues, of which the arterial is one." (p. 130.)

Now this seems to us to be only another and more complicated manner of stating, what every one knew before, that certain impressions would produce the condition termed inflammation. It is the *modus operandi* of these impressions that we have to seek for; and the only step, if it be one in reality, which Dr. Macartney has made, is in attributing their action to their influence on the ganglionic nerves, a doctrine by no means novel. The term *proximate cause* does not seem to us rightly used by him to designate what is at most but the channel through which impressions act on the organic system. It is the change in that system *primarily* resulting from their action, and giving rise to the other more sensible alterations, that properly deserves the title; and on this Dr. M. does not seem to us to have thrown any light. He has, indeed, obscured it rather than otherwise; for we cannot but think that his mode of reasoning on the nature of inflammation is essentially defective, in considering only the state of the vessels, and in leaving out of view the changes to which they are subservient. But is it true that the impressions which produce inflammation necessarily act through the nervous system? We think not. To prove this it must first be shown that the normal changes which constitute the organic functions, all of which are due to the influence of external agents on the organism, depend upon its influence; a doctrine of which we have heretofore shown the instability. If a normal stimulus can produce a healthy change or action, without the intervention of nervous agency, it is perfectly evident that an abnormal stimulus may produce a morbid change independently of it. The remark of Mr. Palmer upon this question strikes us as peculiarly judicious. "The office of the nerves in inflammation appears to hold precisely the same relation to this action that it does to the other organic functions of the body. It is regulative but not essential." (p. 329, *note.*) It is to be remembered that Dr. Macartney's theory involves only the ganglionic nerves, and it is somewhat difficult, therefore, to disprove it; but it is for him to bring forwards the evidence in its favour, of which we can find but little. It is, of course, no objection to his theory, that parts of which the cerebro-spinal nerves are paralyzed are still liable to inflammation; and even more so than in the perfect state, since their nutrition is less active than it should be, and their susceptibility of injury from external agents therefore increased.

"In these cases of paralysis," says Dr. M., "the nerves of the arteries which are chiefly derived from the sympathetic system, have their functions unimpaired; and, therefore, these vessels are influenced by irritations, of which the individual takes no cognizance. I cannot doubt that if the arteries could also, by any experiment, be deprived of their own nerves, or if the connexion of these with the sympathetic were interrupted, none of the phenomena of inflammation would arise, whatever injury might be inflicted." (p. 134.)

We, on the other hand, do very much doubt Dr. M.'s doctrine, and we shall find that it is based on a very insecure foundation. Our readers will recollect that, in his introductory chapter, he maintained that inflammation is liable to occur in different beings, in proportion to the development of their nervous system, and that it does not take place in those which are destitute of that system, namely in vegetables. We took occasion, when adverting to that subject, (Vol. VII. p. 429,) to correct this error, and to show that a state corresponding with inflammation may be produced in plants; and we think this quite a sufficient disproof of the doctrine, that the nervous system is *essentially* concerned in it. There can be little question that by this system are produced, in great measure at least, those sympathetic effects which take place in the system at large from any local disease; and we find these more strongly marked, as Dr. M. has very justly stated, in proportion to the development and complex distribution of this system. But we are by no means warranted in thence inferring that the primary changes are themselves dependent upon it.

A much more satisfactory view of the nature of inflammation, because more practical and comprehensive, is given us by Dr. Carswell; though we think it still open to some of the objections which we have urged against Dr. Macartney's. Although he regards it as in part a disease of the function of innervation, he fully recognizes the change in the vital properties of the blood, and in its actions with the affected tissue, as primarily and essentially concerned. His account of the phenomena, which may be regarded as of constant occurrence, and as in fact constituting the disease, is so good that we shall quote it almost in full. After speaking of the unsatisfactory results of the numerous experiments on the state of the capillary circulation in inflammation, if these be indiscriminately contrasted, he thus proceeds:

"If, however, we carefully examine in detail the numerous experiments which have been made on this subject by Thomson, Wilson Philip, Hastings, Kaltenbrunner, Wedemayer, and Gendrin, and separate the effects of chemical or other agents which are known to modify the tissues and the blood in a manner very different from that of a mere stimulus, from those produced by this latter cause, we shall find that the results have almost always been similar in kind under similar circumstances, and in perfect accordance with the known laws of the vital manifestations of sensibility and contractility—properties, the sensible modifications of which, if not the most important, are the first that occur in the series of changes which constitute the state of inflammation. Equally important, because constant changes are also those which take place in the temperature of an inflamed part, and in the vital and physical conditions of the blood, and, as immediate consequences of these, in the functions of secretion, absorption, and nutrition." (p. 2.)

We are ourselves disposed to believe, for the reasons already hinted at, that the organic functions are those *primarily* affected by such stimuli as produce an inflammatory action; and that the alteration of the *sensibility* (by which we mean, with Dr. C., *conscious* sensibility,) is consequent upon that change. The lesion of sensibility is that which may first manifest itself; but it does not thence follow that it is the cause of the other phenomena. We shall proceed, however, with Dr. Carswell:

"I shall consider inflammation as a disease, the essential phenomena of which present themselves in two successive stages or periods, each of which is referrible to opposite conditions of the physiological properties or functions of the affected part. Although these opposite conditions are sufficiently characterized by the changes which take place in the temperature of the inflamed part, and in the functions of circulation, secretion, and absorption, they are more especially so in those which occur in the sensibility and contractility. The first effect of mechanical irritation is an increase of the sensibility, soon amounting to pain, even in those tissues which, under ordinary circumstances, possess this property in a very low degree; and subsequently, or at the same moment, contraction, or an increased development of the contractile power, if not of the capillaries, of the arteries with which they immediately communicate. After a certain time, varying with the violence of the exciting cause, the sensibility diminishes or ceases to be manifested, for pain is no longer the consequence of the same cause. The contractility undergoes a similar change; the minute arteries cease to contract when stimulated, and they, as well as the capillaries and minute veins, are permanently dilated and distended with blood. That these two opposite states of the sensibility and contractility exist in inflammation, and occur in the order in which I have stated, might, independently of the evidence of the facts themselves, have been believed and understood, for they are but an exaggeration of the law of vital action, or of the physiological conditions of all parts endowed with these properties; a state of activity and repose, of increase and diminution, being the opposite and invariable consecutive conditions of both. It is hardly necessary to observe that these properties, both pathologically and physiologically considered, admit of these opposite conditions in so far only as the exciting cause acts in the strict sense of a stimulus; for if the agent to the influence of which they are subjected is of such a kind as to destroy, or, as is commonly said, to exhaust, instead of increase, there is at once produced a diminution, cessation, or extinction of both, and consequently of their visible signs, such a state cannot, therefore, be regarded as a state of inflammation, for the first or active conditions of the disease have not existed. It is, however, a state which is frequently produced in experiments made to ascertain the effects of stimuli on the capillary circulation in inflammation, and then becomes an interesting illustration of the fact just stated; for although congestion takes place in the part thus debilitated, it is soon followed by the active phenomena of inflammation, or those which characterize its first stage, and a more rapid and extensive formation than usual of those of the second." (pp. 2, 3.)

These remarks are, we think, peculiarly valuable; presenting, as they seem to do, the true explanation of the contradictory phenomena which have so much perplexed pathological observers. We fully accord with Dr. C. in regarding the first effect of a simple stimulus, as an exaltation of the vital actions (whatever may be their character) of the tissue to which it is applied; and Dr. Macartney seems to have lost sight of the fact that this exaltation is manifested in a contraction of the arteries distributed in the part, which does not yield to dilatation until a certain interval has elapsed which varies under different conditions. This exaltation or exaggeration of the regular vital actions of the part is manifested, as we shall presently see, in other ways also; and it is obvious that it differs but little from the condition which we have already discussed under the name of "determination of blood." The effect of certain stimuli in immediately depressing these actions, and thus producing congestion, will recall to the minds of our readers what we then said of the character and causes of that state.

With regard to the changes which are witnessed in the movement of the blood simultaneously with those already described, Dr. C. states the well-known fact that, "in the first stage of inflammation, the circulation is accelerated, and a greater quantity of blood than natural passes into the capillaries; in the second stage, the circulation becomes impeded, and the blood which distends the capillaries ceases at last to circulate." (p. 3.) Dr. C. allows to the altered diameter of the capillaries some influence on the rapidity of the stream of blood through them; but considers as of the greatest importance the relation of the two states to the primary lesions, which implies, he justly remarks, "opposite states of the agents which effect or regulate the transmission of the blood in the capillary vessels." We shall return to this point after enumerating the other most important changes which take place in this disease, which we shall do in Dr. C.'s language.

"During the first stage, the vital properties of the blood undergo a manifest increase. A greater quantity of fibrine is formed, the plastic property of which is increased; for, besides its rapid organization under favorable circumstances, it retains, when separated from the other constituents of the blood, its fluidity for a longer period, and contracts more firmly than in the natural state. The unwonted vigour of the circulation in general, the increased temperature of the whole body, and the resistance opposed to the means employed in inflammation to weaken the vital powers, may also be regarded as connected with this state of the blood, or as inordinate effects of its vital agency through the medium of the nervous system. In this stage too, the temperature is variously and greatly increased; the function of secretion is also for a time augmented; in glandular organs, however, only at the commencement; in serous tissues, for a much longer period, and to a much greater degree. Absorption, if not increased, manifests its activity by the speedy effects of poisons locally employed in this stage.—During the second stage of inflammation, changes, the reverse of those just described, occur in rapid succession. The blood ceases to circulate, coagulates, and assumes a dark colour; the temperature sinks; and secretion, absorption, and nutrition are finally interrupted. If those conditions of the affected part are maintained for a certain time, new products are formed, or other diseased states are produced, as softening, suppuration, ulceration, and mortification, which are therefore denominated *terminations* of inflammation, and constitute separate objects of investigation." (p. 4.)

Dr. Carswell subsequently points out that these opposite conditions may be recognized also in the character of the effusions from the inflamed tissues.

"The different degrees of fluidity, viscidity, and coagulability of the secretions of inflamed tissues are derived from the presence of the serum, albumen, and fibrine of the blood in various proportions. But the most important circumstances connected with these changes in the secretions of inflamed tissues is the formation or separation from the blood of two fluids of an opposite kind, viz. coagulable lymph and pus; the former possessing vital properties, assumes, as it is said, *spontaneously*, but no doubt in virtue of these properties, the solid form, becomes organized, and fulfils the all-bountiful purposes which nature has assigned to it in the economy of life,—the reparation of those injuries so frequently the consequences of the diseases of which it is the product; the latter, possessed of no plastic properties, being, as it were, the residue of the former, and of the molecular structure of organs, by a disorganizing or destructive process, is essentially inert, and like all substances incapable of being assimilated, is separated or removed from the body. The presence of these two products, therefore, marks not only the existence, but serves to distinguish the two most important periods of inflammation. Nor does this apply to the local conditions only of the disease; it is equally conspicuous in the general phenomena which accompany each period, or, as we have said before, each stage of inflammation, by the increased energy or vital power manifested in the first, and the diminution or perversion of the same in the second, and by the nature of the remedies required in each." (p. 12.)

From the consideration of these and other facts, we are led to the belief that the *first* effect of a stimulus calculated to produce inflammation, is on the general organic properties of the part, and especially on its power of deriving from the blood the materials of its assimilating or secreting processes; and that the influence it exerts on the caliber of the vessels, and on the motion of the blood through them, is altogether *secondary* to this. Considering it fully proved that the capillary cirlation is greatly affected by the energy of healthy reaction between the fluid and the tissues it permeates, we cannot doubt that any morbid alteration of this will greatly influence the movement. Accordingly we find that the effect of a stimulus which increases, for a time, the physiological or normal actions of any part, is to accelerate the capilary circulation, whilst the caliber of the vessels is diminished. The latter alteration can scarcely be due to the direct application of the stimulus; but is rather, we think, to be referred to the influence of the ganglionic nerves, which unquestionably are largely concerned in the subsequent processes. To the same influence we should be disposed to refer the simultaneous dilatation of the trunks leading to the part, so as to admit an increased supply of blood, which dilatation, as we have already pointed out, is probably due to the increased nutrition of their elastic coat.

The changes which take place in the blood itself may be accounted for in two ways. We may believe, with much reason, that the physical and vital properties of the whole mass are altered by its passage through the vessels of the affected part, whose parietes are capable of thus influencing it. This idea would correspond with the facts disclosed at a later stage of the process by microscopical examination, as to the evident changes which take place in the blood during its passage through an inflamed part; and it would seem to derive some confirmation from the circumstance that the buffy coat seldom shows itself on blood drawn very shortly after the commencement of inflammation; but that a period of about twenty-four hours seems usually necessary, in man at least, for its development, during which the whole of the circulating fluid will have been repeatedly subject to this influence. On the other hand, it may be argued with much plausibility, that the increase in the quantity and vital properties of the fibrine is due to a sympathetic alteration in the process of sanguification communicated through the ganglionic system: and this view would appear supported by the fact that the buffy coat is commonly found on blood drawn during pregnancy,—a state in which the active assimilating processes which are taking place in the fœtus, may be supposed to require a corresponding amount of the organizable part of the blood, and in which no influence of the maternal vessels on the blood can be supposed to produce the change; as also by the detection of other analogous changes (the formation of pus-globules, for example,) in the blood, when no local cause can be assigned for them. Perhaps we shall not be wrong in assuming that both these causes may be in operation, with varying ratio, in almost every instance.

Whilst the tendency to organization in the blood is thus increasing, the vital properties of the inflamed tissues are undergoing a contrary change. Their power of separating certain elements of the blood, whether for their own nutrition, or for secretion, is impaired; being, it would seem, not merely diminished, but perverted. Hence, the organizable portion of the blood, not being appropriated, as it should be, is effused either into the substance of the tissue or from its surface, as the case may be; and there it may undergo the process of organization by its own inherent properties, requiring only the neighbourhood of a living tissue, to bring it into communication with the general structure, and to supply it with continued nutrition. On this part of the subject we cannot here enlarge, although it is among the most interesting on which we might dwell. We must stop, however, to notice the curious fact brought to light by Dr. Carswell, that the effusion of tubercular matter closely resembles that of coagulable lymph which does not possess sufficient vitality to become organized. And in this connexion, we may introduce some remarks made by Dr. Macartney upon the scrofulous diathesis, which we think very discriminating, and worthy of attention.

"In scrofulous persons, the vascular system is weak, the vessels are small, the blood is deficient in quantity, and is, I think, imperfectly organized; it wants the full power of generating coagulable lymph. . . . . The brain is pale, and does not possess the usual quantity of red blood; and yet there is a mental character belonging to the scrofulous habit, which more strikingly indicates the peculiar state of the constitution than all the other signs. Scrofulous children, in general, exhibit no mental energy, but a gentleness and amiability of disposition, a refinement and judgment in matters of taste, and a purity of moral feeling, which is sometimes so remarkable, as to place them in these points far beyond the scale, and even beyond the conception of the mass of mankind." (p. 82.)

This description strikes us as being more correct than many we have seen. Mental superiority is often dwelt upon as a characteristic of the scrofulous habit; but we agree with Dr. M., that it is rather in the *moral* than the *intellectual* faculties, that the difference is presented.

To return, however, to inflammation. When the change in the vital properties of the affected tissue has proceeded to a considerable length, it affects in a corresponding manner that of the blood which circulates through it; and instead of the organizable lymph, we have an unorganizable product, pus, of much lower degree of vitality, separated from the fluid. The observations of Gendrin on the conversion of the globules of blood into those of pus, by undergoing decolorization and other changes, have obtained currency amongst pathologists; but the late researches of Mandl,* on this point, seem to prove that this account is not correct. He found that when blood was mixed with pus, the red particles of the former underwent disintegrating changes, but did not come to resemble pus-globules; whilst, on the other hand, the latter closely resemble the irregular globules which are formed in the liquor sanguinis, when separated from the red particles, and coagulating imperfectly.

Although the full exposition which we have thought it right to give of our views on the essential nature of inflammation has already extended to such a length, we cannot dismiss this department of our subject without adverting to the highly valuable remarks of Dr. Carswell on the changes which are produced in the tissues themselves by the existence of this morbid action. It would be quite superfluous in us to attempt to criticise such statements from one whose authority on all questions of Pathological Anatomy is so high; and we shall, therefore, content ourselves with such an abstract of his observations as may be interesting and useful to those who have not access to this most valuable work. Excluding the *physiological* characters of inflammation—those deduced from observation of the *actions* of the part,—the physical or *anatomical* characters consist in modifications of its natural colour and vascularity, and of its consistence and bulk. Of these, the most conspicuous are increased redness and vascularity; but as these may occur in other states besides that of inflammation, it becomes necessary to seek for some diagnostic indications whereby this may be recognized. The *primary* or most general forms of inflammatory redness may be described as the *ramiform*, *capilliform*, *uniform*, *punctiform*, and *maculiform*.

"The first and second of these, as the terms imply, have their seat in the small arteries and veins, and in the capillaries respectively; the third and fourth are produced by capillary injection, the uniform red colour which accompanies the former being the consequence of an accumulation of blood in the entire capillary system of a part; the minute dotted or punctiform redness of the latter arising in the peculiar

* Anatomie Microscopique, liv. ii.

structure of the part, as in the villosities of mucous membranes, when the seat of inflammation, apart from the mucous tissue itself. The maculiform has also its seat in the capillary vessels, is sometimes produced by the accumulation of blood being greater in some points than others, but much more frequently by rupture of these vessels and extravasation, and hence may be called the *hemorrhagic* form of inflammation. Exclusive of the influence of various modifying causes, but more especially of the quantity and quality of the blood, the several degrees of inflammation are expressed by each of these forms in the order in which they are enumerated, the ramiform indicating the least, the maculiform the greatest degree." (p. 5.)

Uniform redness is best seen in dense vascular tissues, such as the skin, where it always occurs as the first physical sign of inflammation: in inflammation of mucous membranes, however, it is always preceded by the capilliform or punctiform injection ; and in these, also, the ramiform is remarkably conspicuous, whilst the maculiform or hemorrhagic is more frequent than in any other tissue. "All these forms, indeed, varying in degree, are met with combined in the mucous membranes, more especially of the digestive organs; the capilliform and punctiform being the first that appear, and the most characteristic of inflammation." In all the serous membranes, uniform redness is produced in the same manner; but ramiform injection never occurs in them,—really having its seat, where it appears to be present, in the subjacent cellular tissue. Indeed, adds Dr. C., "it has always appeared to me that even the capilliform injection of these membranes is in great measure produced by the penetration of the blood into their softened substance by the *vis à tergo*. I have certainly never been able to discover blood-vessels in the arachnoid, in inflammation of the pia mater, except where it lies in contact with this membrane." The same source of fallacy must be guarded against in other cases, since the red colour which any membranous tissue presents may be owing to blood accumulated in the subjacent cellular substance, the hue of which is transmitted through it, according to its degree of transparency. In parenchymatous organs, inflammatory redness is strongly marked; but from their complex structure, and the great quantity of blood with which they become impregnated during life and after death, from various causes, it is often extremely difficult to say how far the redness depends on this circumstance or on capillary injection. In organs naturally of a pale colour, redness and the causes of it are sufficiently obvious; it depends on the uniform capillary injection of the first or second stage of inflammation, and is succeeded by the punctiform or hemorrhagic.

The *secondary* forms which inflammatory redness presents, constitute the distinctive local characters of many diseases, especially in the cutaneous and mucous tissues. On these, however, we shall not at present dwell.

Other changes of colour are noticed besides *redness*, especially in the chronic or asthenic states of inflammation. From a more or less complete stagnation of the blood in the capillaries, and from certain changes which it undergoes there, especially when neither suppuration nor effusion of coagulable lymph are taking place, various shades of purple, brown, and black, are produced. When the discoloration is punctiform, as when seated in the villosities of the alimentary canal, it produces a gray or slaty hue, called by French pathologists *couleur ardoisée*.

Most or all of these appearances, however, may be artificially produced in many organs by local circumstances operating either during life or after death, without the existence of inflammation; and it becomes a matter of great importance to be able to distinguish the injection of the vessels produced by gravitation or mechanical obstacle to the return of the venous blood, and the coloration of the tissues by imbibition, from the effects of disease which they so closely resemble.

"The differential characters of inflammation and local congestion are founded on certain differences in the physical characters of each, and in the circumstances under which they respectively occur. Although similar forms of redness and vascularity are produced in both, it is perhaps only in mucous membranes that a difficulty arises in distinguishing the one from the other. So long as the redness and vascularity are confined to the capillary vessels, or have their seat in the villous or follicular structure, there can be no doubt as to their inflammatory nature. It is only when they become more general and present the ramiform character that any difficulty arises. This, however, is removed by an examination of the neighbouring veins, which, in mechanical congestion, will be found dilated, tortuous, or even varicose, according to the degree and duration of the obstacle by which it has been caused. The congestion of the veins may likewise be traced to its cause. In inflammation, the local congestion commences in the capillaries, afterwards extends to the small veins, but never to large branches; in mechanical congestion, the blood accumulates first in the trunks, which are always conspicuous, and afterwards in the branches and capillaries. It is only when mechanical congestion is combined with inflammation, that the anatomical diagnosis becomes difficult or impossible." (*Carswell*, p. 9.)

No distinction of this kind, however, will hold good with regard to the vascularity produced by the *depending* position of parts during life or after death, which perfectly resembles that of inflammation, when occurring in the same parts. Our determination must be grounded, therefore, upon the presence or absence of this cause.

The redness observed in tissues in contact with the blood after death, produced by *imbibition*, and also that occasioned by transudation when decomposition has taken place, are easily distinguished, according to Dr. C., from that which is the consequence of inflammation.

"It can never be confounded with that of inflammation, when it occurs along the course of the larger subcutaneous veins; but it has frequently been so in the lining membrane of the large arteries especially, and of the veins. Whatever may be the circumstances which favour the red colour of imbibition, it never bears a near resemblance to that of inflammation. It is a mere die of a uniform, almost scarlet, red colour, generally limited to the lining membrane, without any other perceptible change of the coats of the vessel; whereas redness from inflammation is of a dull, rather pink, tint, extending more or less to the other coats, accompanied by a fine capillary injection of the subjacent cellular tissue, and marked congestion of the vasa vasorum; the lining membrane is softened and opaque or easily removed; the cohesion of the other tunics is diminished; they are also thickened or swollen, and infiltrated with serosity or coagulable lymph." (p. 10.)

The *permanence* of the redness and vascularity of inflammation after death is regarded by Dr. C. as a character by which alone, and under doubtful circumstances, we may ascertain the existence of this disease, and distinguish it from the local congestions with which it may be confounded.

"The redness and vascularity of mechanical congestion, of position or gravitation, and of imbibition, differ, as we have seen, essentially from those of inflammation, in the mode of their production and other local circumstances. But they also differ essentially in other respects. Thus, all these kinds of redness and vascularity are produced and maintained by appreciable causes, which operate external to the vessels, without the blood or the vessels themselves undergoing any change, by which the vital properties of either are so modified as to render the redness or vascularity permanent after death. By means of ablution, pressure, or scraping, both of these physical characters disappear in a short time. In inflammation it is far otherwise; the employment of the same means never removing, or effecting only a slight diminution of either. Injections, too, which penetrate the capillaries in the former kinds of congestion, cannot be made to reach them in that which proceeds from inflammation. It is necessary, however, to observe, that this state of the capillaries, and the redness which accompanies it, and which constitutes what I mean by the *permanency* of these physical characters of inflammation, are more or less decided by the degree and stage of the disease. In the highest degree or second stage, the permanency of both is most marked; in the first stage it is much less, the redness diminishing or disappearing entirely after death, in slight inflammations of the skin of short duration. This difference in the permanency of the redness and vascularity of inflammation in its two stages may be explained by the facts already noticed, viz., the absence of coagulation of the blood in the first and its occurrence in the second stage, in which also the fibrine unites with and penetrates the capillaries, thereby retaining the colouring matter of the blood, and producing occlusion of the vessels. Although, however, I have said that *redness* disappears after death, in slight inflammation of the skin, some degree of increased *vascularity* remains, as is seen by comparing the diseased with a healthy part. And, besides, it is of great importance to know that, even in such slight cases of inflammation, the cutis undergoes changes which render the existence and extent of the disease very conspicuous after the disparition of the redness, and from one to two days after death. The affected parts only of the skin assume a purplish tint, and become infiltrated with bloody serosity, and the epidermis is detached from these parts much sooner than from those which were not affected by the inflammation. This post-mortem congestion, and a more rapid tendency to decomposition than in ordinary circumstances, are conditions which ought not to escape the notice of the pathologist, when it occurs in internal organs. They are sometimes the only morbid appearances which are met with in fatal cases of scarlatina, and especially in rubeola, and indicate the extent not only of the inflammation, but of the depressing influence which it must have exercised on the vital function of these organs." (*Ib.* pp. 10, 11.)

The practical importance of these remarks must be our apology for not having abridged them. Every man of experience must have met with cases in which the difficulty of distinguishing the marks of inflammation from the results of other agencies has been both perplexing and important; and this is more especially the case in medico-legal investigations. There can be no doubt that in past times the existence of the marks of inflammation has often been too hastily credited; and that the erroneous inferences, founded upon such belief, have led even to the sacrifice of innocent lives. Perhaps in later times the tendency has been to err in the other extreme, by not admitting the previous existence of inflammation, unless some of its peculiar products are discoverable. Any diagnostic marks which can aid us in drawing a correct middle line are evidently, therefore, of great importance; and, without saying that Dr. Carswell has done all that could be wished in establishing these, we are satisfied from his remarks, that the difficulty is by no means incapable of being overcome.

Our determination may be assisted, as already hinted, by careful observation of the state of consistence of the affected part. Acute inflammation almost invariably produces *diminution of cohesion* between its organic elements; and this change, which of course arises from the depravation of the nutritive processes, is apparent in many cases of inflammation where redness and vascularity have disappeared, or mark but faintly the degree of alteration which the disease has effected in the process of nutrition. The solidification of spongy organs during acute inflammation, as when the lungs are hepatized, might appear a fact contrary to this general doctrine; but this solidification is only due to the retention of the effused fluid in the tissues; and although they feel harder than natural when compressed, the diminution of cohesion which has taken place between their anatomical elements is rendered conspicuous by the facility with which they are penetrated, broken down, and crushed. "The necessity and importance of recognizing this physical character of inflammation must therefore be obvious, as it not only enables us to detect the existence and appreciate the degree of this pathological state, but also to recognize the first and most simple organic alteration to which it gives rise." An opposite condition, a state of *induration*, is a frequent consequence of inflammation when chronic, or, at least, often accompanies it. It differs from the solidification in acute inflammation in this, that there is at the same time increased cohesion of the anatomical elements of the affected part. It is obviously the consequence of the organization of the coagulable lymph effused in the first or second stages of acute inflammation, either into the interstitial cellular structure of organs, or into the tissues themselves, in the state of softening just described.

The increase of *bulk* which accompanies acute inflammation, and which depends upon the accumulation of blood in the part and the retention of effused fluids, is an alteration which acquires importance rather from the effects to which it gives rise than from its value as a physical character of acute inflammation.

Every one knows the old definition of inflammation—*rubor, tumor, cum calore et dolore;* and, having discussed the two former, we should advert to the latter, had we anything either novel or peculiarly important to say regarding them. It is not part of Dr. Carswell's plan to treat of them, and Dr. Macartney is more than usually mystical upon the subject. He quotes the experiments of Brodie, as if their fallacy had never been exposed, to show that animal heat does not depend upon respiration; and considers that we are to look upon animal heat as "one of the phenomena of sensibility." We do not see how this can be regarded as a step in the enquiry. There can be no doubt that its evolution is greatly influenced by the nervous system; but, as we conceive, only through the medium of those molecular changes to which it is immediately due, and which are without doubt controlled to a certain extent by the agency of that system. Two facts mentioned by Dr. M. are new to us, and of considerable interest. He states that if the principal nerve of an extremity be divided, the member becomes instantly three, four, or five degrees hotter than it was before; and that division of the spinal marrow produces an immediate elevation of the temperature of the posterior limbs. "A case," he adds, "has been related to me of death by injury to the spinal marrow, near the head, in which the heat of the body rose as many as twelve degrees (if I recollect right) above the natural standard." (p. 13.) This effect he considers due to the liberation of the organic actions from the restraints under which they are carried on as long as the influence of the individuality of the nervous system is maintained. We, on the other hand, should be disposed to regard it as due to the temporary excitement of the molecular changes by the irritation produced by the section of the nerve, and propagated to its extremities: just as a similar irritation will, in many cases, produce muscular movements. If Dr. M.'s doctrine were true, the temperature of a paralyzed limb would be *permanently* hotter than that of the sound one, which everybody knows not to be the case. We are not, therefore, disposed to accord with him in the belief that the increased heat of inflamed parts is to be ascribed "more to their state of local or organic sensibility, than to the condition of their arteries, as regards circulation and secretion," (p. 15;) since we are fully convinced that it is in the altered state of the nutritive processes that we are to look for one cause of the high temperature—the increased quantity of blood sent to the part being another. It is a well-known fact that when a portion of the surface of the body is inflamed, the *respiration* (so to speak) of that portion—in other words, its conversion of the oxygen of the air in contact with it into carbonic acid,—is increased; and this, harmonizing with what we know from other sources of the relation between the disengagement of carbon from the body and the maintenance of its temperature, seems to us nearly decisive on the question.

When speaking of the *pain* of inflamed parts, we find Dr. M. using the term *sensibility* in its ordinary sense; a change of meaning which, we fear, will be rather puzzling to many of his readers. He very properly points out that the pressure of distended vessels, the tumefaction of the tissues, &c. aggravate the pain, but do not produce it; since the same degree of pressure or tension would not cause pain in parts not inflamed. We are disposed to think with him that the increased quantity of blood sent to the part will of itself augment the *impressibility* of the nervous matter, since we know how intimately its functions are connected with the activity of the circulation of the vital fluid through it.

In regard to the *treatment* of inflammation laid down by Dr. Macartney, we do not find much that calls for particular remark from us, except the *rationale* which he assigns for the effects of the various remedies which he recommends. The following is his classification of these:

"1st. Remedies which diminish the force of the heart, and give the disposition generally to the small arteries to go into the contracted state. 2d. Means that effect a diminished size of the arteries, or reduce the sensibility in the inflamed part. 3d. Medicines that augment or reproduce the natural secretions, and thereby abate the circulation, or lessen the effusions made into inflamed parts. 4th. Counter-irritants, secretions, or impressions made in different parts from those which are inflamed. 5th. Lotions or fluids which exert sedative and astringent power. 6th. Means for affecting in an agreeable manner the sensations of inflamed parts. 7th. Causes which produce an easy or satisfied state of feeling, on the sentient surfaces, or in the individual." (p. 143.)

Under the *first* head Dr. M. of course ranks general bleeding, the intention of which "is to produce that kind of impression which is followed by a weaker action of the heart, and a more contracted state of all the smaller arteries in the body." This is doubtless true; but it is by no means the whole truth. Here and elsewhere Dr. M. has entirely passed over the changes which the vital properties of the blood undergo in this disease, and which are modified by remedial means perhaps even more than the action of the heart and the state of the vessels. The latter, indeed, being but secondary to the local changes of which we have formerly spoken, cannot be the essential object of treatment, except when it is found impossible to reach the affected part but by an impression made upon the whole system. This impression is, we think, rather communicated by the nervous system, and by a change in the circulating fluid itself, than by any alteration in the action of the heart and arterial tubes. When blood is suddenly abstracted, "the nervous system," to use Dr. M.'s rather peculiar language, "takes alarm at the rapid departure of the vital fluid; the heart beats feebly, and the arteries also sharing in the sense of danger, are disposed to contract, even beyond the degree which would be necessary to accommodate their tubes to the reduced volume of the fluid in circulation." The effect of loss of blood upon the properties of the circulating fluid itself is essentially that of lowering its vitality, as evidenced by its more rapid and at the same time less firm coagulation; and the want of harmony between the properties of the fluid and those of the solids with which it reacts is thus diminished: The influence of nauseating medicines is exercised, according to Dr. M., in the same manner as that of abstraction of blood, operating only in diminishing the force of the circulation. This, we must repeat, is but a very limited view of their efficacy. They lower the whole tone of the system, and diminish the vital activity of every part. Hence it is in the first stage of inflammation that they are most useful. The *contra-stimulant* plan of administering tartar-emetic is thus summarily dismissed: "It has become fashionable of late to give and repeat large doses of this remedy, on what grounds I cannot understand, as small doses are sufficient to produce all the effects we wish for, provided they be repeated with sufficient frequency."

Of the remedies of the *second* class, which effect a diminished size of the arteries, or reduce the sensibility of the inflamed part, *topical bleeding* is among the chief. We quite agree with Dr. M. that the rationale commonly assigned to its action—that of its directly and mechanically diminishing the quantity of blood circulating through the inflamed part, is a very fallacious one; and yet we cannot see that he has done much towards its elucidation by telling us that it operates by creating "the organic consciousness of vacuity, which always produces the tendency to contraction." Considering, as we do, that the dilated state of the capillaries is but secondary to the condition of the assimilative processes, we must look for the efficacy of this and other local remedies in its influence upon the latter. How they act it may not be easy to determine; but how much more do we know of the action of any external agents upon the system? Among the local applications to inflamed parts, those which *abstract temperature* are ranked by Dr. M. as of the highest consequence. We have already seen that, by the regulation of temperature, the supervention of an inflammatory state may be often prevented, and that the reparation of injuries may thus be effected in a manner more resembling that of simple natural growth. By the same means a considerable degree of control may be exercised over the inflammatory state of parts accessible to their application, when this has already commenced; but they must be used with judgment to be successful.

"The reduction of the heat of any part of the body in an *extreme degree* renders the existence of inflammation impossible in that part during the time; but as cold is a direct sedative to all vital actions, there is a temporary suspension also of the process of reparation. The remedial operation of a *moderate degree of cold* is in most cases to be preferred. It diminishes in place of extinguishing sensibility and vascular action, and under its operation the reparative processes can be carried on." (p. 157.)

It is in repressing the increased action with which the *first* stage of inflammation commences that we should *à priori* look for the greatest efficacy of *cold,* since it is well known to be a sedative to vital action in general; and we think that experience fully bears out this principle. Having formerly noticed pretty fully the advantages of cooling applications, and the method of using them recommended by M. Josse, we shall not again travel over the same ground. It may be advantageous, however, to quote Dr. M.'s mode of employing *irrigation,* as a very simple and easily-managed one.

"The most easy and manageable way of employing irrigation is to place the limb of the patient in a trough, and having laid some lint on the inflamed part, to let the water be conducted by means of a strip of woollen cloth, from a vessel holding the water or other fluid, which may be placed on a chair or table standing beside the bed. One end of the strip is to be inserted into this vessel; the other, which should be cut into a pointed shape, laid on the lint. The water will then proceed in the manner of a syphon continually from the vessel, not by drops falling from a height, the sensation of which is disagreeable. The water is carried off by a tube proceeding from the end of the trough into a vessel placed at the end of the bed. I have found that a strip of cloth of some breadth where it is inserted into the water, and ending in a point where it touches the lint, answers the purpose of a syphon much better than the filaments of candle-wick which some surgeons have employed. The patient with this apparatus is able to vary his position, which is a great comfort to him. It is obvious that irrigation can only be used with convenience to the extremities. The water may have any degree of temperature that is desired; and if it should be wished to employ iced water, the vessel holding it may be placed at distance from the patient's bed, or even outside the room, and conveyed by an elastic tube on which there is a cock, to regulate its admission into a smaller vessel, situate near the bed." (p. 159.)

Dr. Macartney lays considerable stress, and we doubt not with justice, on the propriety of applying and withdrawing cold *gradually* when an intense degree is used. This can only be proper in cases of very severe injury, where the inflammation cannot be restrained by any other means. Where the inflammation is not very violent, a moderate degree, such as that obtained by irrigation, is generally suitable, especially in summer and in warm climates. "A very simple rule," he adds, "may be safely followed with regard to the use of cold applications: which is, to consult the feelings of the patient. Wherever they alleviate the pain, they do good; and wherever they have not this effect, they are improper." It is certainly very provoking to find, as the practitioner so often does, that the application on whose efficacy he most relied must give place to one of an opposite character, from its uncongeniality to the feelings of the patient; and, while we fully agree with Dr. M. that these may be taken as our safest guide, we would also point out that the very circumstance of our being obliged to rely upon them shows that we have yet much to learn upon the subject, and that we must not neglect the consideration of any of the phenomena of diseased action, however insignificant they may appear. Having, in a former article, adverted to Dr. M.'s claims as the reviver of the remedial employment of cold water, we need not discuss these again. We hope that the publication of these statements regarding its efficacy, which his oral prelections have already diffused pretty extensively, will contribute to the more extended employment of a means of cure, the extraordinary efficacy of which in some of the most dangerous circumstances is only paralleled by its extraordinary simplicity. With the following quotation we shall conclude what we have to say of this interesting subject:

"In a letter from Dr. Bourgen, a very intelligent pupil of mine, now settled in Demerara, received the 23d of June, 1837, he states that a medical man in extensive practice there uses water-dressing after amputation and other operations; and that these wounds healed as well as in the best-treated cases in cold climates; and that in fourteen amputations he had performed, he had not lost a single patient by tetanus." (p. 193.)

We do not find anything under the *third*, *fourth*, and *fifth* heads, which calls for especial remark, except that Dr. M. has found the *constant* application of the lead-lotion an infallible remedy for *tinea capitis*, curing even cases of several years' duration in as many days. The means by which *an agreeable state of feeling* is produced are those on which he lays most stress for the abatement of the "sense of injury" consequent upon dangerous accidents, and for the encouragement of the reparative processes, which will effect a cure when sources of irritation are removed. The use of *fomentations* is popularly known to have this effect to a certain degree; but Dr. M. has found the employment of *steam* at moderate temperatures to be far more efficacious, so as almost to deserve the character of a new remedy. Of this we have already spoken when treating of the reparative processes in our former article; and we must again refer to Dr. M.'s work for the details of its employment. The application of *water-dressing* is also ranked by him under this head; and we are glad to perceive that it is gaining ground among English surgeons, possessing, as it does in most instances, so many advantages over the common poultice. Until, however, their physiological notions are so far reformed that they can conceive that a wound may heal more advantageously without granulation and suppuration than with them, we cannot hope to see it employed as extensively as it deserves.

As to the classification of these remedies in Dr. M.'s arrangement, we must take leave to say that it appears to us even less correct than that of others which we have already criticised. The "state of feeling" of any part, whether its "sensibility" be of the conscious or unconscious kind, must depend upon the nature of the actions going on in it; and though it may indicate the normal or abnormal character of those actions, we cannot conceive how remedial applications can affect it primarily, or otherwise than through them. Remedies of this class, therefore, we should rather consider as having an immediate tendency, like others, to restore the natural actions of the part, and *thence* to produce "an agreeable state of feeling."

Dr. Macartney lays much stress, and with great propriety, upon "freedom from the sense of restraint, pressure, and friction, an easy and elevated position, and avoidance of all motion," as remedies for inflammation. Though all surgeons and patients would assent to the propriety of the above circumstances being procured, both surgeons and patients are too often practically remiss in their attention to this part of the treatment. Knowing, as we do, the extent to which a state closely resembling inflammation in many of its characters may be induced by the mechanical conditions only of the part, we cannot wonder that when this morbid action is established, it should be aggravated and maintained by such conditions, if they are allowed to remain. We must again remark, however, that the sensations produced by them are only the indications of their existence; and that it is not, therefore, the *feelings* which are to be treated, as Dr. M. seems to suppose, but the conditions which give rise to them.

With one more observation we shall conclude our review of Dr. Macartney's work. He notices at the end of it the experiments of Dr. Guyot, on the treatment of wounds by dry, heated air, a plan whose results are obviously conformable to his own doctrine of the modelling process. Some experiments on the treatment of amputations in this manner have been lately published, from which it would appear that, if the cure be not accomplished more rapidly, it takes place with less constitutional disturbance than in the ordinary manner. From the experiments of Dr. Guyot, joined to those of Dr. Macartney, it would seem well established that the temperature of the animal is the degree most favorable to the simple reparative processes by which breaches of substance are filled up by natural growth without inflammatory action. Whether dry air or steam is the best application for this purpose is a point well worthy of investigation; and we would strongly recommend the trial of both to such of our readers as have opportunities of putting them in practice. We shall now dismiss Dr. M.'s treatise, which, with all its faults, we regard as a valuable contribution to pathological science and to the healing art.

We had intended concluding this article with a pretty full analysis of the *Teoria della Flogosi* of Signor Rasori; but as our allotted space has been already occupied by subjects which we deem more important, we shall notice at present only a few points of special interest. Our general appreciation of the value of the work has already been given;* and as we have adverted in the same place to the analogies and differences between the leading doctrines of its author and those of Brown, it will be sufficient to remind our readers that he recognizes *two* diatheses as the cause of diseases; and that, while he views *debility*, requiring the administration of wine, opium, and other stimulants, as very frequently concerned, he also regards the *diathesis of stimulus* as the cause of all inflammatory diseases, and as requiring the most vigorous antiphlogistic treatment, combined with the use of those remedies which he denominates *contra-stimulant*, from their supposed power of controlling or repressing excitement without giving rise to any increase in the evacuations.

The views of Rasori, on the subject of the buffy coat, will not, we suspect, meet with the sanction of many observers in this country; yet, perhaps, they may show that further enquiry is still requisite to determine its true conditions. He affirms that its presence is to be considered in all cases a decisive proof of the existence of inflammation, and believes that its quantity is in all instances accurately indicative of the degree of

* Vol. vii. p. 426.

inflammation. To the validity of this test he admits of no exceptions. The appearance occasionally exhibited, by the blood drawn from pregnant women, cannot, he asserts, be mistaken, by any experienced practitioner, for the true inflammatory coat, as it consists at the most in a thin, soft, muciform layer, scarcely differing in colour from serum, and unaccompanied by any of that firmness or cupping characteristic of a true buff. In all cases where a true coriaceous covering appears, he asserts, that inflammation must exist somewhere, however latent it may be. He seems, however, entirely to overlook the fact that it is possible to superinduce the true buffy coat, even on healthy blood, by the influence of repeated venesection. Another instance of similar hasty generalization is his assertion that, whenever there is an absence of the characteristic marks of inflammation after death, no inflammation could have existed during life, even for a short time. We apprehend that this dogmatical statement could not well stand the test of certain cases of rapidly-fatal erysipelatous affections either of the skin or mucous membranes.

His idea of the essential nature of inflammation is so obscure that we do not pretend to understand it. He maintains that the character of an inflamed part consists in congestion of the *venous* capillaries; but that this results from a general increase of action in the arterial system, the source of which is to be found entirely in the heart. His idea, if we apprehend it aright, seems to be that all inflammation depends on a generally increased action of the heart and arteries, the local effect or increase of vascularity being determined upon some spot in those portions of the body most liable to inflammation, where the arterial capillaries happen to be somewhat more crowded than in the adjacent parts, and thus bear rather more than the average proportion to the venous capillaries in which they terminate. The blood being now introduced into the latter with more than the usual force, and in more than the ordinary quantity, in consequence of the above-mentioned increase of arterial action, they become, as a matter of course, morbidly dilated. Thus an universal "diathesis of stimulus," to use Rasori's own phrase, would seem to be presupposed, as an indispensable preliminary to the local effect. On this point, however, his disciples are very far from implicitly following their master. Tommasini, in particular, has taken great pains to make it known as his belief that very many general diseases, fevers, &c., have, on the contrary, their origin in the propagation of some local morbid excitement. The insufficiency of Rasori's hypothesis discovers itself, we think beyond dispute, when applied to inflammations originating in an obviously local cause, as, for instance, mechanical or chemical irritants.

Inflammation, he asserts, never gives rise to organized products. As for the vessels occasionally seen in the morbid exudations of lymph on serous membranes, they are by no means new formations, but merely some of the preexisting vessels of the membrane displaced, and pushed from their original nidus by the secretion of fibrine beneath them! He denies also, that ossification is ever the result of inflammation, in respect to which, as well as the previous assertions, we need hardly remind our readers that he stands in direct opposition to some of the highest authorities in morbid anatomy.

He animadverts strongly on the application of the term *weakness* to that state of vessels which exists in the chronic stage of inflammation; and asserts that such of the so-called astringents as are known by experience to be useful in this condition, as, for example, in the latter stages of conjunctivitis, really owe their efficacy to their contra-stimulant virtue. As for opium, he bestows on it his unmeasured condemnation in all such cases; for, according to his theory, it is, and can only be under any circumstances, a stimulant, and nothing but a stimulant. Thus, like a thoroughgoing theorist, he makes all things, even facts themselves, bend to his hypotheses.

Amongst the greatest peculiarities of his system is the opinion, which he looks on as indubitable, that groups of symptoms, in all respects similar, as far as we can perceive, may arise in connexion with the opposite diathesis, a circumstance which will, of course, give rise, in many instances, to no small embarrassment as to the kind of treatment proper to be pursued. He seems in such dubious cases to renounce all further attempts at diagnosis founded on rational or physical signs, and to have recourse at once to experiment. Thus, if in an affection apparently of an inflammatory character, antiphlogistics have already been pushed to what he thinks the extreme length of safety, without benefit, he considers it a sufficient proof that the true diathesis of the malady has been mistaken; he reverses his plan, and puts the stimulant practice into full operation, beginning with moderate doses, and gradually but promptly raising them to heroic ones. Opium, ether, and wine are thus employed with as free a hand as Brown himself could have desired, and with perfect confidence of success. He gives, in an appendix, two series of cases, the first consisting of diseases treated as inflammatory by a free employment of antiphlogistic measures, till the patients were brought to the very brink of the grave, and yet eventually saved on the diagnosis being happily reformed, and an opposite line of treatment (wine and opium) liberally adopted; the second comprising cases in which the erroneous diagnosis being persisted in to the end, and copious bloodlettings and other lowering methods employed throughout, a fatal termination was the result; and here, on examination, the dead body exhibited no marks of true inflammation, simply because none had ever existed. Our practical readers will be at no loss to discover a different *rationale* for the success of the alteration in the mode of treatment in the first class of cases; since every one knows that when diseases, unquestionably inflammatory in character, have been kept in check by rigorous antiphlogistic measures, a period is very likely to supervene, in which the exhaustion of the system requires support, whilst the subsidence of the inflammation permits its administration. It is no proof of the incorrectness of the diagnosis that such should be the case, though experience has too often shown that there are members of our profession unworthy enough to take advantage of such a circumstance to raise their own reputation at the expense of that of others. The second class of Rasori's cases does not seem to us to prove anything, except upon the supposition, to the fallacy of which we have already adverted, that inflammation, existing during life, always leaves evidence of its operation in the dead body; and although errors in diagnosis will, in the present state of our knowledge, be sometimes made by the most accomplished physician, they are far from justifying Rasori's assertion, that the same group of symptoms may present themselves in totally opposite states of the system.

We shall now bring this subject for the present to a close, hoping that, however imperfect our outline may have been, it will at least serve to exhibit the more important results of modern observation, to develope the bearing of physiological doctrines, and to indicate the questions which most require elucidation. The activity with which almost every branch of enquiry into the operations of life is now being carried on leaves us no doubt that we shall, at no very distant period, have reason to recur to this topic; indeed, we hope ere long to embody some important observations on one department of it, the formation of pus, in a connected view of the progress of our knowledge regarding the physical conditions of vital action.

